# Calcified Pituitary Adenoma Mimicking Craniopharyngioma: A Case Report

**DOI:** 10.7759/cureus.54352

**Published:** 2024-02-17

**Authors:** Fahad B Albadr, Anwar H Alhatlani, Nawaf S Alhelal, Abdullah A Albakri, Ahmed A Alhumidi, Mohammed A Alshwieer

**Affiliations:** 1 Radiology and Medical Imaging/Neuroradiology, King Saud University Medical City, Riyadh, SAU; 2 Medicine, Qassim University, Buraydah, SAU; 3 Medicine, King Khalid University Hospital, Riyadh, SAU; 4 Medicine, King Saud Hospital, Unaizah, SAU; 5 Pathology, King Saud University Medical City, Riyadh, SAU; 6 Family and Community Medicine, King Saud University Medical City, Riyadh, SAU

**Keywords:** macroadenoma, visual fields, adenoma, craniopharyngioma, neuroimaging, calcification, pituitary

## Abstract

A 60-year-old woman presented with a history of a previously diagnosed sellar mass and a recent onset of severe headache, vision loss, and dizziness. The patient was found to have a large mass with curvilinear calcification on imaging. Histopathology confirmed the presence of a pituitary adenoma with abnormal acini, consistent with adenoma, and moderate amounts of granular eosinophilic cytoplasm. A detailed analysis of the patterns of calcification and the radiological morphology is crucial to distinguishing between pituitary adenoma and craniopharyngioma. Recognition of these patterns can aid in distinguishing between these conditions, providing a more accurate diagnosis and an effective treatment plan.

## Introduction

Pituitary adenomas are the most prevalent tumors found in the sella region, constituting roughly 5% to 20% of all central nervous system tumors. In terms of imaging, pituitary adenomas rarely display peripheral thick calcification; the presence of calcifications often indicates other sellar region masses, such as craniopharyngiomas, meningiomas, and aneurysms, rather than adenomas [1]. Histological examinations suggest that calcification, particularly the formation of psammoma bodies, occurs in about 15-25% of pituitary adenomas [2], although this is quite rare in radiological findings, which account for only 0.2-8% of cases [1].

Calcification in pituitary adenomas typically appears in one of two forms: (1) an "eggshell" appearance, where the adenoma is encased by a thin layer of calcification, potentially indicating an expanded sella or a calcified pseudocapsule, or (2) multiple small calcification nodules within the adenoma. The term "pituitary stone," however, refers to a large, dense calcium deposit within a pituitary adenoma [3-5].

In this study, we present a case of a patient with a sellar mass displaying curvilinear calcification, which is more indicative of craniopharyngiomas. Distinguishing between adenomas and craniopharyngiomas is of paramount importance due to their varying management strategies.

## Case presentation

A 60-year-old female patient presented to the clinic with a progressive headache, rapidly worsening vision over the past month, and occasional dizziness. She had been diagnosed with a sellar mass 15 years ago and had chosen not to undergo surgery. Hence, she was treated medically. Her past medical history included a single incident of a transient ischemic attack treated with aspirin.

**Figure 1:**
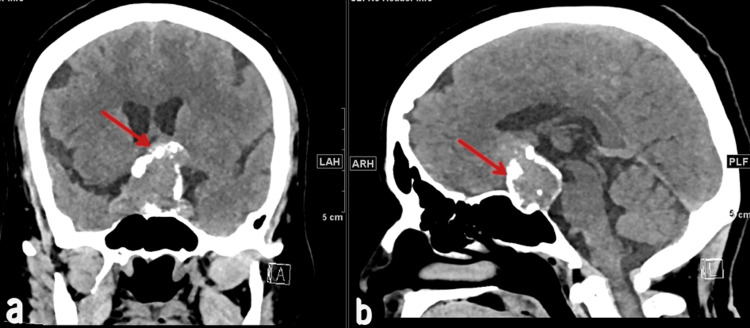
A non-contrast-enhanced brain CT scan showing a pituitary mass with curvilinear peripheral thick calcification in coronal (a) and sagittal (b) views, indicated by red arrows

**Figure 2:**
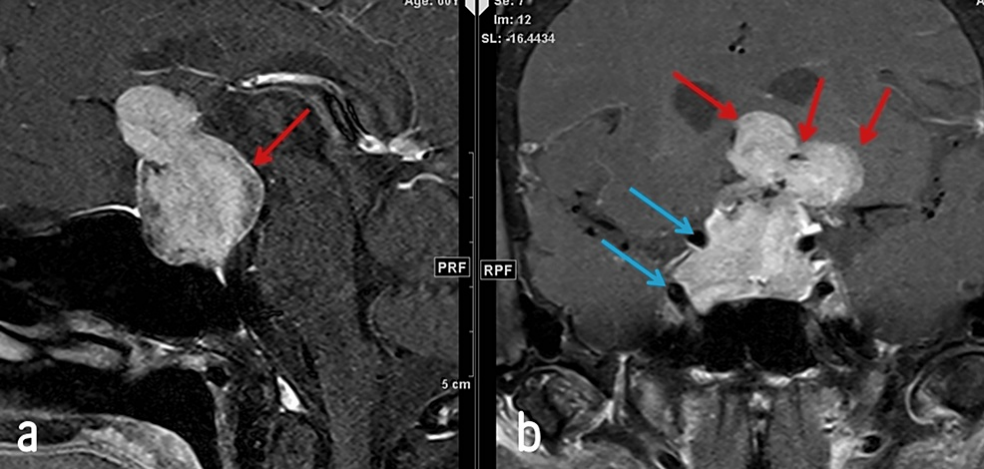
Enhanced T1-weighted fat-saturated brain MRI in both sagittal (a) and coronal (b) views revealing a mass causing significant compression on the hypothalamus and optic chiasm (red arrow). Additionally, an encasement of the internal carotid arteries is observed without any narrowing or occlusion (blue arrows). No cystic component is detected

**Figure 3:**
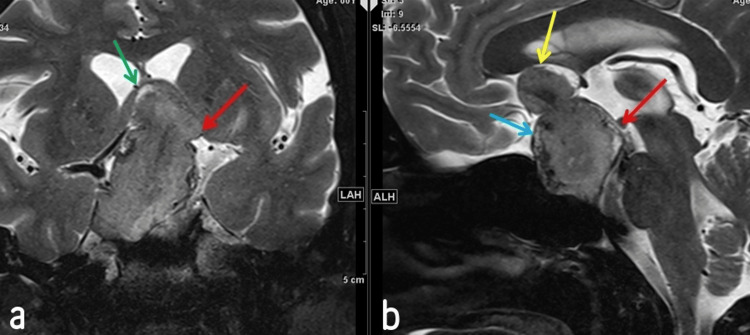
T2-weighted MRI in both coronal (a) and sagittal (b) views. These images reveal a sellar and large suprasellar mass with an intermediate signal intensity that elevates the third ventricle. The mass elevates the rostrum of the corpus callosum (yellow arrow). Furthermore, it abuts the mamillary bodies (red arrow). Peripheral calcification is also evident (blue arrow). Lastly, there is an elevation of the fornix (green arrow)

Physical examination revealed left temporal hemianopia and right superior quadrantanopia, with no other notable abnormalities. Routine blood tests showed hyperprolactinemia. Preoperative scans, including a non-contrast enhanced brain CT scan (Figure 1) and T2-weighted brain MRI (Figures 2-3), were conducted. The brain CT scan revealed a sellar mass with curvilinear peripheral thick nodular calcification. The MRI demonstrated a large lobulated solid lesion filling and expanding the sella turcica with suprasellar extension. The masa had an intermediate T2 signal along with slightly heterogeneous postcontrast enhancement. The lesion measured 28 x 32 x 45 mm in anteroposterior, mediolateral, and craniocaudal dimensions, respectively.

**Figure 4:**
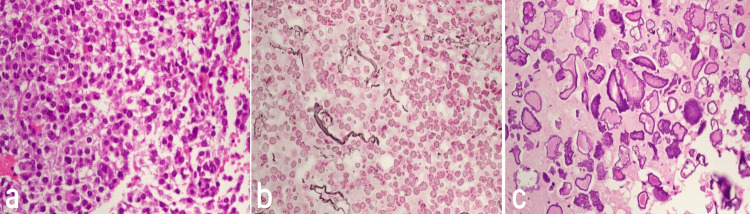
Photomicrograph of the tumor revealing round to polygonal cells with abundant eosinophilic to clear cytoplasm focally forming acini (a). Reticulin stain reveals partial loss of reticulin fibers (b) (reticulin stain, original magnification x 400). Other areas show an abundance of calcification (c) (hematoxylin and eosin stain, original magnification x 400)

Histopathology of the sample displays pituitary tissue composed of unevenly enlarged acini. These acini consist of sheets of uniform cells with small, round nuclei, moderate amounts of granular eosinophilic cytoplasm, and extensive calcification (Figure 4).

An immunohistochemistry study was conducted on the specimen. Reticulin stain reveals abnormal disruption of the acini, consistent with adenoma. The cells within the lesion are strongly positive for synaptophysin. Some tumor cells exhibit positive results for growth hormone and prolactin, although these are relatively rare. Thyroid-stimulating hormone is focal and weak. Follicle-stimulating hormone and luteinizing hormone also show weak positivity, but these are also relatively rare. The cytokeratin marker is negative. The tumor tissue is negative for S100. The Ki-67 proliferative index shows a focal increase (10%). All the serum levels of the pituitary function profile were within normal ranges.

A final diagnosis of pituitary adenoma was established through radiological morphology and calcification patterns and confirmed by the histopathological examination. Subsequently, the patient underwent successful treatment with endoscopic endonasal trans-sphenoidal resection of a macroadenoma. Following the procedure, the patient exhibited stability and marked improvements in visual fields and diplopia. However, we did notice an increase in polyuria and elevated serum sodium levels, along with low urine osmolality and specific gravity during urinalysis, indicating a confirmed diagnosis of central diabetes insipidus. To manage this condition, we administered desmopressin on a fixed dose schedule twice daily.

## Discussion

Intracranial calcifications detected through a CT scan can either be physiological or related to age, or they may be pathological. Pathological calcifications might occur due to genetic disorders, infections, inflammatory diseases, neurotoxicity, vascular disorders, intra-axial neoplasms, or extra-axial neoplasms such as meningiomas or craniopharyngiomas [6]. Each condition presents a distinct pattern of calcification on radiological imaging.

Radiological evidence can help distinguish between sellar and suprasellar lesions based on various calcification patterns, such as curvilinear, nodular, or mixed. This helps identify the type of lesion [7]. Common masses originating from the suprasellar region include pituitary adenomas and craniopharyngiomas.

Craniopharyngiomas often have a lobulated shape with multiple cystic and solid parts [8]. Calcification is common in these cases, accounting for about 90% of all craniopharyngiomas [8]. Tumoral calcification is particularly seen in 70% to 90% of childhood craniopharyngiomas and 40% to 60% of adulthood tumors [9]. Pituitary adenomas, on the other hand, can show different CT attenuation patterns depending on their components. Various non-contrast attenuation can be seen in solid, cystic, hemorrhagic, or necrotizing components [10], but calcification is less common in these cases, reported in only 0.2% to 8% of instances [10].

Intratumoral calcifications are crucial in identifying suprasellar lesions, as they can help narrow down the differential diagnosis to conditions like craniopharyngioma, aneurysm, chordoma, and cartilaginous tumors. These calcifications are best displayed using CT scans and usually don't show any signal on spin-echo MRI [11,7]. For instance, craniopharyngiomas can be identified by a solid component with its own calcification and nodular enhancement within a mixed cystic-solid multiloculated structure; these features are considered pathognomonic for craniopharyngiomas [12].

Other differentials include cavernous malformation, where calcification is present in about 50% of cases with popcorn kernel resemblance. Aneurysms are rare and possible radiological differentials, despite their clinical features. They present with a well-defined lobe-shaped calcified formation in the suprasellar or parasellar region [13,14]. Rim calcification may be present with giant aneurysms. Calcification of associated dural structures is also suggestive of meningiomas and, in particular, tuberculum sella meningiomas. A meningioma, unlike a pituitary adenoma, is an extra-axial, dural-based mass that shows hypointense or isointense gray matter on T1-weighted MRI images and hyperintense or isointense on T2-weighted images. On CT, the meningioma appears as an extra-axial mass displacing the normal brain, adjacent to dural structures, and sometimes calcified or multilobulated [15].

The surgical method chosen for pituitary adenoma depends on factors like suprasellar extension, tumor size, and the degree of calcification [16]. Traditionally, the trans-sphenoidal surgical approach has been utilized for these cases [16-18]. However, in the case reported by Ibrahim et al., a craniotomy was performed due to extensive calcification [19]. Another case by Murase et al. demonstrated the successful removal of the tumor using the endoscopic endonasal approach, suggesting that this method can also be viable for treatment [20]. Transcranial surgery is typically recommended for patients with adenomas with prominent extrasellar extension, adenomas with extensive fibrosis, those who have failed trans-sphenoidal surgery, or those with inadequate decompression and uncertain diagnoses. Additionally, the presence of ectatic carotid arteries, severe sinus infection, or aneurysms may necessitate an open craniotomy [15].

## Conclusions

In this case report, we discuss a patient who has been dealing with a known sellar mass for a long period. This individual presented with mass-effect complications and symptoms; a diagnosis of pituitary adenoma was made, which was further confirmed by radiological imaging showing the presence of curvilinear calcification.

Recognition of the pattern and morphology of calcification for both sellar and suprasellar masses can help distinguish between a pituitary adenoma and a craniopharyngioma. Our detailed analysis of the preoperative images, which included patterns of calcification, the absence of a cystic component, and solid enhancement of the sellar and suprasellar mass, suggests that the patient likely has a pituitary adenoma rather than a craniopharyngioma.
